# Pan-European Study on Functional and Medical Recovery and Geriatric Rehabilitation Services of Post-COVID-19 Patients: Protocol of the EU-COGER Study

**DOI:** 10.1007/s12603-021-1607-5

**Published:** 2021-03-08

**Authors:** Stefan Grund, M.A.A. Caljouw, M.L. Haaksma, A.L. Gordon, R. van Balen, J.M. Bauer, J.M.G.A. Schols, W.P. Achterberg, COGER-Study Subgroup of the EUGMS-SIG on GR

**Affiliations:** 1 Center for Geriatric Medicine, Agaplesion Bethanien Hospital Heidelberg, Geriatric Center at the Heidelberg University, Rohrbacher Straße 149, 69126, Heidelberg, Germany; 2 Department of Public Health and Primary Care, Leiden University Medical Center, Leiden, the Netherlands; 3 Division of Medical Sciences and Graduate Entry Medicine, University of Nottingham, Derby Medical School, Royal Derby Hospital, Derby, UK; 4 NIHR Applied Research Collaboration-East Midlands, Nottingham, UK; 5 Department of Health Services Research, -Focusing on Value-based Care and Ageing-and Department of Family Medicine, Caphri — Care and Public Health Research Institute, Maastricht University, Maastricht, The Netherlands

**Keywords:** COVID-19, older persons, post-acute, geriatric rehabilitation, ADL functioning

## Abstract

**Objectives:**

There is insufficient knowledge about the functional and medical recovery of older people infected with SARS-CoV-2. This study aims to gain insight into the course of functional and medical recovery of persons who receive geriatric rehabilitation (GR) following SARS-CoV-2 infection across Europe. Special attention will be paid to the recovery of activities of daily living (ADL) and to the GR services offered to these patients.

**Design:**

A multi-center observational cohort study.

**Setting and participants:**

This study will include several European countries (EuGMS member states) each providing at least 52 comparable routine datasets (core dataset) of persons recovering from a SARS-CoV-2 infection and receiving geriatric rehabilitation. The routine data will be anonymously collected in an online CASTOR database. The ethical regulations of each participating country will be followed.

**Primary outcome:**

ADL functioning.

**Secondary outcomes:**

Length of stay, discharge destination, hospital readmission and mortality. Other variables that will be collected are quality of life, treatment modalities, complications, cognition, frailty, mood/anxiety, BMI, nutrition and pain. All variables will be reported at admission and compared with follow-up scores (discharge, 6 weeks and 6 months follow-up).

**Conclusion:**

This study will explore the effect of geriatric rehabilitation on post-COVID-19 patients, especially on ADL recovery, and the variety of geriatric rehabilitation services across Europe. Information from this study may help improve recovery of older persons infected with SARS-CoV-2 and improve geriatric rehabilitation services in the ongoing COVID-19 pandemic.

## Introduction

The COVID-19 pandemic is hitting the world hard. Older people over the age of 70 years are more likely to get very ill when infected with SARS-CoV-2. The majority of patients admitted to the hospital with COVID-19 are in this age group. They have the highest morbidity and mortality all infected people. Some of them need intensive care, including ventilatory support. COVID-19 is often associated with prolonged hospitalisation and sequelae include multi-organ failure, pulmonary dysfunction, physical deconditioning, chronic fatigue, sarcopenia and pressure ulcers. There is also a high incidence of mental problems following COVID-19 including cognitive decline, Post-Traumatic Stress Disorder (PTSD) and Post-Intensive Care Syndrome (PICS). (1, 2, 3, 4) To regain functional independence, many of these patients will need specialised rehabilitation.

Because COVID-19 is a new illness, with unique constellations of symptoms affecting multiple body systems (5), we do not know how the course of recovery for patients will be, or what treatment approaches will deliver the best outcomes. We do know from early experience in Italy, that the rehabilitation process is difficult, and the course is capricious (6, 7).

Geriatric rehabilitation is a multidimensional approach of diagnostic and therapeutic interventions, the purpose of which is to optimise functional capacity, promote activity and preserve functional reserve and social participation (8). Patients in geriatric rehabilitation have specific needs associated with the ageing process. For example, they have complex health issues including disabilities due to pre-existing conditions and geriatric syndromes such as frailty, cognitive impairment, and sensory loss. Geriatric rehabilitation is often delivered in parallel with acute geriatric care (9) which could be an advantage in post-COVID-19 patients, as periods of clinical instability, which require more intensive input by acute care teams, may be a feature.

It is known that infections are associated with deterioration in performance of activities of daily living (ADL) among vulnerable older people who had no ADL restrictions before the infection (10). So, it can be assumed that older people who have been infected with SARS-CoV-2 will also have limitations in ADL functioning and need special attention to support independence. One of the goals of geriatric rehabilitation is to enable older people to manage their ADL without the assistance of another person or to minimize the need for external assistance (11).

A number of COVID-19 patients have required intensive care admission, therefore rehabilitation experiences from other ICU patients may be useful as we learn how to rehabilitate those with COVID-19 (12, 13, 14). Rehabilitation principles applied in Post-Intensive Care Syndrome (PICS) may apply in addition to specialist pulmonary rehabilitation pathways currently used in older COPD patients (15). However, we are currently in the dark as to what the clinical characteristics of post-COVID-19 patients admitted for GR are, what treatment they receive, and what functional and clinical outcomes they have after geriatric rehabilitation. There is insufficient knowledge about the course of their disease and their functional and medical recovery.

The members of the Special Interest Group for Geriatric Rehabilitation of the European Geriatric Medical Society (EuGMS) designed this study to get insight into the course of functional and medical recovery in older persons affected by SARS-CoV-2 receiving geriatric rehabilitation across Europe.

Therefore this study aims to: 1) explore the course of ADL recovery and influencing factors; 2) describe other outcomes after geriatric rehabilitation in post-COVID-19 patients and; 3) describe geriatric rehabilitation services provided to post-COVID-19 patients across Europe.

## Methods

### Design and study population

An international multi-center observational cohort study. During one-year (October 2020 – October 2021), patients will be enrolled in the study in geriatric rehabilitation facilities of EuGMS members across Europe.

The study will be conducted according to the principles of the Declaration of Helsinki (2013 version) and in accordance with the General Data Protection Regulation (GDPR) and in full conformity to any applicable state or local regulations in the participating countries.

The study population will consist of older people rehabilitating after a SARS-CoV-2 infection in a geriatric rehabilitation setting in one of the participating European countries.

### Inclusion criteria

• Recovering from a SARS-CoV-2 infection, confirmed with Polymerase Chain Reaction for viral RNA, serology for antibodies against SARS-CoV-2, or alternative tests as they become established and nationally mandated by individual governments.
• Accepted into a geriatric rehabilitation service, either institutionally-based or provided at home.

### Exclusion criteria

• Presence of severe cognitive impairment, which leads to insufficient decisional capacities to participate in the study.
• Subjects who did not give informed consent, or who have opted out of using their anonymous data for research purposes where opt-out is an option.

### Recruitment and consent

Eligible participants will be recruited in sites for geriatric rehabilitation in several countries, of which Germany, the Netherlands, Belgium, Switzerland, United Kingdom and Russia have already expressed intention to participate. In these sites, post-COVID-19 patients admitted for geriatric rehabilitation will be informed about the study and asked if they object to the use of their anonymized regular care data for research purposes to improve geriatric rehabilitation after COVID-19 infection. If they do, the data will not be used (optout procedure). In some countries this will be conducted under the guidelines for service evaluation and audit, as it uses routine data, held entirely by the clinical care team. Where this is not possible because of local legislation or guidelines, informed consent will be obtained. We propose to exclude participants who lack the mental capacity to consent to participation in research or service evaluation, as arrangements for inclusion of such participants are highly variable between countries. Such participants are unlikely to be common in geriatric rehabilitation facilities in most countries and we do not think, therefore, that our sample will be significantly biased by their exclusion.

### Geriatric Rehabilitation settings and treatment

The settings in which geriatric rehabilitation is provided are heterogenous across EuGMS countries. In the Netherlands geriatric rehabilitation is mainly provided in, or run by, nursing homes, in the United Kingdom it can be provided in intermediate care facilities or community hospitals, and in Germany, Belgium, Switzerland and Russia it takes place in geriatric rehabilitation facilities or in special rehabilitation/geriatric wards in the hospital. In several of these countries it can also be provided in the home environment with the support of specialist teams.

Participants will receive standard rehabilitation treatment according to the discretion of clinical teams, which will likely be adapted to the specific needs of post-COVID-19 patients. This treatment includes physical therapy, occupational therapy, and medical treatment by an advanced nursing practitioner, geriatrician or medical specialist. Additional ad hoc input by other therapists, including but not limited to speech and language therapists, dieticians, orthotists and podiatrists, will be captured. This study will not influence any therapy provided or decisions about the medical treatment or treatment programs already used in the participating geriatric rehabilitation setting.

### Outcome measures

#### Primary outcome

The primary outcome measure is: ADL functioning.

ADL functioning will be assessed with the Barthel Index, Utrecht Scale for the Evaluation of Rehabilitation (USER) or Functional Independence Measure (FIM), depending on use in the participating country. The USER (16) and FIM (17, 18) will be converted afterwards to the Barthel Index using standardised approaches.

#### Secondary outcomes

The secondary outcomes are separated into functional and medical outcome measures and rehabilitation descriptors (19).

#### Functional and medical outcome measures

Additional functional and medical outcomes include: complications, nutritional status (including Body Mass Index), performance of mobility/balance, muscle strength, fatigue, dyspnea, cognition, mood, pain, quality of life, post-traumatic stress syndrome and mortality

#### Rehabilitation descriptors

General rehabilitation descriptors are length of stay, discharge destination, and hospital readmission. GR service descriptors are kind of professionals involved, and kind of treatment given.

### Data collection

For each participating geriatric rehabilitation facility in Europe one instructed local care professional will complete an online CASTOR database (20) in which anonymous data from the clinical records of the included patients will be collected at admission, discharge and 6 weeks and 6 months follow-up (see table 1: data collection scheme). These data concern:

**Table 1:** Data collection scheme

**Variable**	**Admission**	**At discharge**	**Follow-up**
*Participant characteristics*			
Year of birth/age	X		
Gender	X		
Residency premorbid	X		
*Hospital/general practitioner*			
Hospital admission and number of days hospital admission	X		
Stay at ICU and days at ICU	X		
Complications during hospital stay	X		
Complications at home	X		
COVID-19 diagnosis confirmed (PCR/Serology)	X		
*Geriatric rehabilitation*			
Pre-morbid ADL functioning (2 weeks prior to hospital admission)	X		
ADL functioning	X	X	X
Frailty pre-admission (2 weeks prior to hospital admission)	X		
Frailty	X		
Body Mass Index before COVID-19 infection	X		
Body Mass Index	X	X	
Nutrition	X	X	
Comorbidity	X		
Fatigue	X	X	
Dyspnoea	X	X	
Pain	X	X	
Quality of life	X	X	X
Complications during GR stay		X	
Length of stay		X	
Discharge destination		X	
Cognition	X	X	
Mood/anxiety/depression	X	X	
*By indication/optional*:			
Swallowing problems	X		
Speech problems	X		
Timed up and Go test	X	X	
MRC biceps/quadriceps	X	X	
Hand grip strength	X	X	
Post-traumatic stress syndrome			X


ICU=Intensive Care Unit; ADL=Activities of Daily Living; PCR=Polymerase chain reaction; GR=Geriatric Rehabilitation; MRC=Medical Research Council scale

a. Demographic data: Year of birth/age, gender, comorbidity, and premorbid residency of the participants.
b. Prior information from hospital or general practitioner (GP): number of days of hospital admission, number of days at the ICU, complications during hospital stay or at home (Thromboembolism, Delirium, Pressure Ulcer), and SARS-CoV-2 diagnosis confirmed by PCR or Serology.
c. At admission and discharge in geriatric rehabilitation: in all participants, a core dataset, which includes the primary and secondary outcome parameters, will be collected. See table 1 for an overview of these outcome parameters. These data are already routinely collected in regular geriatric rehabilitation. At discharge we will additionally collect information from the treatment plan about which professionals have been involved during geriatric rehabilitation and which treatment interventions have been applied.
d. Follow-up: at 6 weeks, 6 months following regular care in the participating countries: ADL functioning, quality of life, and post-traumatic stress syndrome.

### Core data set and data homogenization

As shown in Table 2, the assessments used for the routine care are very heterogeneous among the participating countries. In order to be able to obtain comparable data, the core data set of the study is homogenized (see Table 3).

**Table 2:** Assessments used for routine care in the countries

**Domain**	**Variable**	**NL**	**DE**	**UK**	**RU**	**CH**	**BE**
ADL	ADL functioning	USER; Barthel Index	Barthel Index	Barthel Index	Barthel Index	FIM	Barthel Index
Quality of life	EQ-5D-5L	EQ-5D-5L	EQ-5D-5L	EQ-5D-5L	EQ-5D-5L	EQ-5D-5L	EQ-5D-5L
Frailty	Frailty	Clinical Frailty Scale	Clinical Frailty Scale (translated German Version)	Clinical Frailty Scale	Clinical Frailty Scale	Clinical Frailty Scale	Clinical Frailty Scale
Nutrition	Body Mass Index (BMI)	BMI	BMI	BMI	BMI	BMI	BMI
	Malnutrition	SNAQ65	NRS/ MNA	MUST	MNA	NRS	MNA
Comorbidity	Comorbidity	wFCI	wFCI	wFCI	wFCI	wFCI	CIRS-G/ wFCI
Cognition	Cognitive impairment	USER	MMSE/ Demtect	MOCA	MMSE/ MOCA	MMSE/ MOCA	MMSE/ MOCA
	Delirium	DOS	4AT	4AT	4AT	SQiD	DOS, 4AT
Mood/anxiety	Depression	USER	GDS-15	GDS-15	GDS-15	GDS-15	GDS-15
Pain	Numeric rating scale (NRS-P)	NRS-P	NRS-P	NRS-P	NRS-P	NRS-P	NRS-P
Skin	Pressure ulcer: yes/no	Yes	Yes	Yes	Yes	Yes	Yes
symptoms	Fatigue: yes/no	Yes	Yes	Yes	Yes	Yes	Yes
	Dyspnoea: yes/no	Yes	Yes	Yes	Yes	Yes	Yes
*Optional*:							
Mobility/balance	Performance	TUG	TUG	TUG	TUG	TUG	TUG
Muscle strength	MRC biceps/quadriceps	MRC biceps/ quadriceps	MRC biceps/ quadriceps	MRC biceps/ quadriceps	MRC biceps/ quadriceps	MRC biceps/ quadriceps	MRC biceps/ quadriceps
Post-traumatic stress syndrome	PTSS	Yes	Yes	Yes	Yes	Yes	Yes
Speech/swallowing	Swallowing: yes/no	Yes	Yes	Yes	Yes	Yes	Yes
	Speech: yes/no	Yes	Yes	Yes	Yes	Yes	Yes


NL=The Netherlands; BE=Belgium; DE=Germany; UK=United Kingdom; RU=Russia; CH=Switzerland; ADL=Activities of Daily Living; USER= Utrechtse Schaal voor de Evaluatie van Klinische Revalidatie; FIM=Functional Independence Measure; wFCI= Weighed Functional Comorbidity Index; SNAQ65= Short Nutritional Assessment. Questionnaire for 65+; NRS= Nutritional Risk Score; MNA= Mini Nutritional Assessment; MUST= Malnutrition Universal Screening Tool; MMSE=Mini Mental State Examination; MOCA= MOntreal Cognitive Assessment; Demtect= Dementia detection test; SQiD= Single Question in Delirium; 4AT= 4 'A's test; DOS= Delirium Observation Screening; HADS= Hospital Anxiety and Depression Scale; GDS-15= Geriatric Depression Scale — 15 items; VAS= Visual Analogue Scale; TUG= Timed Up and Go; MRC= Medical Research Council scale; PTSS= Post-Traumatic Stress Syndrome

**Table 3:** Assessment domains and instruments — Core data set and data homogenization

**Domain**	**Variable**	**Core data set**	**Data homogenization**
ADL	ADL functioning *(Barthel Index, USER, FIM)*	USER; FIM Barthel Index	Conversion into Barthel Index (0–20 pts.)
Quality of life	EQ-5D-5L / EQ-5D VAS	EQ-5D-5L / EQ-5D VAS	EQ-5D-5L (tariff/country), EQ-5D VAS (0–100 pts.)
Frailty	Frailty *(Clinical Frailty Scale)*	Clinical Frailty Scale	Clinical Frailty Scale (1–9 pts.)
Nutrition	Body Mass Index *(BMI)*	BMI (kg/m^2^)	BMI (kg/m^2^)
	Malnutrition *(SNAQ65, MNA, MUST, NRS)*	SNAQ65/ NRS/MNA/MUST	1 = suspicious (MNA ≤ 11 pts., NRS ≥ 3 pts., MUST ≥ 2 pts., SNAQ65 = moderate/high risk) 0 = non suspicious
Comorbidity	Comorbidity *(Weighed Functional Comorbidity Index)*	wFCI	wFCI (0–36 pts)
Cognition	Cognitive impairment *(MOCA, MMSE-2, Demtect)*	USER/ MMSE/ MOCA/ Demtect	1 = suspicious, (MMSE ≤ 27 pts, Demtect ≤ 12 pts., MOCA ≤ 26 pts.) 0 = non suspicious
	Delirium *(DOS, 4AT, SQiD)*	DOS/ 4AT/ SQiD	1 = suspicious (4AT > 3 pts., DOS > 3 pts., SQiD > 0) 0 = non suspicious
Mood/anxiety	Depression *(HADS, GDS)*	HADS/ GDS-15	1 = suspicious (GDS ≥ 6 pts., HADS ≥ 8 pts.) 0 = non suspicious
Pain	Numeric rating scale *(NRS-P)* Pain	NRS-P	NRS-P (0–10 pts.)
Skin	Pressure ulcer: yes/no	Pressure sores query	1 = yes = existing 0 = no = non existing
Symptoms	Fatigue	VAS-scale	VAS-scale (0–10 pts.)
	Dyspnoea	VAS-scale	VAS-scale (0–10 pts.)
Mobility/balance	Performance *(Timed up and Go)*	TUG	TUG (seconds)
Muscle strength	MRC biceps/quadriceps	MRC biceps/quadriceps	MRC biceps (0–5pts.)/quadriceps (0-5pts.)
Post-traumatic stress syndrome	PTSS	PTSS (Yes/No)	1 = yes = PTSS existing 0 = no = PTSS not existing
Speech/swallowing	Swallowing	Swallowing problems (Yes/No)	1 = yes = Swallowing problems existing 0 = no = Swallowing problems not existing
	Speech	Speech problems (Yes/No)	1 = yes = Speech problems existing 0 = no = Speech problems not existing


pts.=points; ADL=Activities of Daily Living; USER= Utrechtse Schaal voor de Evaluatie van Klinische Revalidatie; FIM=Functional Independence Measure; SNAQ65= Short Nutritional Assessment. Questionnaire for 65+; NRS= Nutritional Risk Score; MNA= Mini Nutritional Assessment; MUST= Malnutrition Universal Screening Tool; MMSE=Mini Mental State Examination; MOCA= MOntreal Cognitive Assessment; Demtect= Dementia detection test; SQiD= Single Question in Delirium, 4AT= 4 'A's test; DOS= Delirium Observation Screening; HADS= Hospital Anxiety and Depression Scale; GDS-15= Geriatric Depression Scale — 15 items; VAS= Visual Analogue Scale; TUG= Timed Up and Go; MRC= Medical Research Council scale; PTSS= Post-Traumatic Stress Syndrome

### Sample size calculation

The primary outcome is ADL functioning (Barthel index or derived from the USER or FIM). The Barthel index is recommended to be used as ADL measure for research and care practice in older populations (21). In our study, ADL functioning will be measured repeatedly in each participant. Therefore we performed a power calculation for a paired sample t-test on the primary outcome ADL functioning (Barthel index). We assume a minimal clinically important difference of 2 points on the Barthel index as relevant (22); this is the mean difference between Barthel score at admission and Barthel score at discharge (23, 24). Therefore the study would require a sample size of 52 (number of pairs) to achieve a power of 80% and a level of significance of 5% (two sided), for detecting a mean of the differences of 2 between pairs, assuming the standard deviation of the differences to be 5.

### Statistical analysis

Descriptive statistics will be used to give an overview of characteristics of the participants in the participating countries for all primary and secondary outcomes.

A paired sample student t-test will be used to test the difference between ADL functioning at admission and follow-up per participating country.

Multivariable analyses will be used to evaluate the influence of individual factors (age, gender, comorbidities using modified Charlson comorbidity index (25) and frailty status before COVID-19 infection) as well as GR service factors (rehabilitation team structure, treatment intensity, treatment modalities) on changes in ADL functioning between admission and follow up.

For the other variables (paired) sample student t-tests and Mann-Whitney U-tests will be used for parametric and non-parametric continuous or ordinal variables respectively, whilst or chi-square tests will be categorical variables.

No imputation will be used for missing data. We will report the proportion of missing data for each variable. The level of significance will be set at p < 0.05.

## Discussion

Post-acute care, including rehabilitation, is an important aspect of the recovery of COVID-19 survivors in many countries around the world (26). While most studies and statements of patient rehabilitation that are currently underway focus on adapted conventional rehabilitation in acute care (27) or post-acute care settings (28, 29, 30, 31), the present study examines the outcome and structural adjustments of post-acute geriatric rehabilitation for post-COVID-19 patients.

In contrast to many other current COVID-studies (27, 32), the present study is a multi-center study. The Pan-European approach of this study will enable us to understand the spectrum of geriatric rehabilitation services for post-COVID-19 patients across Europe.

Because geriatric rehabilitation structures are heterogenous in Europe (33), the baseline and outcome measures used in routine practice are also heterogenous. To overcome this barrier a homogenization process of the core data set will be performed in order to remove inconsistencies in interpretation of the cut-off points of the assessments used. Post-acute geriatric rehabilitation services, which includes therapy modalities, and the team members involved will be evaluated and compared between participating countries.

This study will focus on a prospective observation of routine medical and therapeutic care adaptions in post-acute geriatric rehabilitation of the new patient group «post-COVID-19 survivors», excluding patients without COVID-19.

The study will identify specific requirements for and barriers to successful rehabilitation of older COVID-19 survivors. This will help in the future to better prepare and triage these persons for geriatric rehabilitation and to focus on special therapies during COVID-19 geriatric rehabilitation. We will also gain a sense of how geriatric rehabilitation services work across the continent in times of pandemic.

### Strengths and limitations

This is the first study examining COVID-19 rehabilitation on a European level. It uses routine care data and therefore requires minimal time investment of care providers. This study will provide knowledge about the effect of routine post-acute geriatric rehabilitation care on recovery of ADL in older post-COVID-19 persons. In addition, it will provide insight into the variety of geriatric rehabilitation services offered in different European countries during the SARS-CoV-2 pandemic. Differences in routine post-acute geriatric rehabilitation care results and structures between different European countries will be assessed.

A limitation of this study is the observational study design and the likeliness of missing data due to the use of routine care data. Another limitation is that the study does not cover all post-acute geriatric rehabilitation patients. Patients with severe cognitive impairment, which leads to insufficient decisional capacities to participate in the study, will be excluded. Observational studies can measure correlation between exposures and outcomes but cannot imply causality because of the absence of control data. We will, however, be able to make important inferences because of the likely size of the sample and the expected variation in practice between European countries. Due to the use of routinely collected data, inferences from this study will be restricted to the variables collected in routine practice. The choice of the Barthel Index (ADL functioning) as the primary outcome will be affected by the usual limitations of this measure, including ceiling effects (the inability to measure improvements beyond the maximum threshold) and the fact that the Barthel Index is an ordinal, rather than a continuous scale. However, we expect that the Barthel Index is sensitive enough to capture the change in ADL among post-COVID-19 patients.

## Conclusion

This study will explore the effect of geriatric rehabilitation on post-COVID-19 patients, especially on ADL recovery, and the variety of geriatric rehabilitation services across Europe. Information from this study may help improve recovery of older persons infected with SARS-CoV-2 and improve geriatric rehabilitation services in the ongoing COVID-19 pandemic.

### Key summary points

• This study evaluates the course of functional and medical recovery during routine geriatric rehabilitation care in older people affected by SARS-CoV-2.
• This multi-center observational cohort study will include facilities in several European countries and observe functioning in Activities of Daily Living, length of stay, discharge destination, hospital readmission and mortality as outcomes.
• Information from this study may help improve recovery of older people infected with SARS-CoV-2 and the geriatric rehabilitation services offered to them, and also inform development of services beyond the pandemic.
